# Chaperonin GroEL Reassembly: An Effect of Protein Ligands and Solvent Composition

**DOI:** 10.3390/biom4020458

**Published:** 2014-04-22

**Authors:** Nataliya Ryabova, Victor Marchenkov, Nina Kotova, Gennady Semisotnov

**Affiliations:** Institute of Protein Research, Russian Academy of Sciences, Institutskaya Street 4, Pushchino 142290, Russia; E-Mails: ryabova@phys.protres.ru (N.R.); march@phys.protres.ru (V.M.); nina@alpha.protres.ru (N.K.)

**Keywords:** oligomeric protein folding, chaperonin GroEL, denaturation, reassembly

## Abstract

Chaperonin GroEL is a complex oligomeric heat shock protein (Hsp60) assisting the correct folding and assembly of other proteins in the cell. An intriguing question is how GroEL folds itself. According to the literature, GroEL reassembly is dependent on chaperonin ligands and solvent composition. Here we demonstrate dependence of GroEL reassembly efficiency on concentrations of the essential factors (Mg^2+^, ADP, ATP, GroES, ammonium sulfate, NaCl and glycerol). Besides, kinetics of GroEL oligomerization in various conditions was monitored by the light scattering technique and proved to be two-exponential, which suggested accumulation of a certain oligomeric intermediate. This intermediate was resolved as a heptamer by nondenaturing blue electrophoresis of GroEL monomers during their assembly in the presence of both Mg-ATP and co-chaperonin GroES. Presumably, this intermediate heptamer plays a key role in formation of the GroEL tetradecameric particle. The role of co-chaperonin GroES (Hsp10) in GroEL assembly is also discussed.

## 1. Introduction

The study of unfolding and refolding of mature proteins mainly confirms Anfinsen’s hypothesis that all information about the protein spatial structure is encoded in the protein amino acid sequence [1,2]. At the same time, the analysis of vital activity of the cell under stresses reveals a number of proteins called molecular chaperones which are involved in either catalysis of protein folding or regulation of distribution of newly synthesized proteins between competing pathways of protein folding and aggregation [3,4,5]. Besides, the chaperones assist assembly of oligomeric complexes, transmembrane transport of protein chains and their degradation [3,6,7,8,9,10]. Some of the chaperones are oligomeric proteins consisting of many subunits (from 10 kDa up to 100 kDa each) arranged in some cases in one- or two-ring quaternary structures [11,12]. An intriguing question arises as to how chaperones fold. *E. coli* heat shock proteins GroEL (Hsp60) and GroES (Hsp10), usually called chaperonins [7,11,12], are the best studied both structurally and functionally. The chaperonin GroEL is an oligomeric complex consisting of 14 identical subunits (60 kDa each) arranged as two interacting ring-shaped 7-subunit structures [13]. Upon functioning, GroEL interacts with another oligomeric protein-co-chaperonin GroES consisting of 7 identical subunits (10 kDa each) arranged as a dome-like ring-shaped structure [14,15]. GroEL unfolding-refolding studies were initiated more than 20 years ago by Lissin *et al*. [16]. The authors have shown that a complex tetradecameric GroEL particle can be reconstructed (refolded) *in vitro* from its urea-unfolded monomeric state in the presence of Mg-ATP. GroEL renaturation in the absence of Mg-ATP results in folded monomers (subunits) which cannot specifically oligomerize [16]. These folded monomers have a large content of the secondary structure and compactness, but show lower stability than the full-size tetradecameric GroEL (see also [17,18,19]).

An assembly of folded monomers is controlled by several factors that are described inconsistently. For example, in the original work it was demonstrated that GroEL reassembly occurred in the presence of Mg-ATP but not Mg-ADP. Besides, the efficiency of GroEL reassembly increased after addition of either native GroEL (self-chaperonining) or co-chaperonin GroES [16]. However, there are reports on GroEL reassembly in the presence of Mg-ADP and ammonium sulfate [20,21] and even in the absence of adenine nucleotides [19,22,23]. The presence of native GroEL is not obligatory either for oligomerization of its subunits [20,21]. Thus, we conclude that GroEL reassembly is determined by both its ligands and ambient conditions. Here we clarify the role of ligands and solvent composition in GroEL reassembly. Firstly, we show that either at low ionic strength or at low protein concentrations both native GroES and Mg-adenine nucleotides (Mg-ATP or Mg-ADP) are necessary for assembly of GroEL monomers. Secondly, we have studied in detail dependence of the efficiency of GroEL reassembly on concentrations of its ligands (Mg^2+^, ADP and ATP), as well as on concentrations of ammonium sulfate, NaCl and glycerol, to evaluate the minimal concentrations required for initiation of GroEL reassembly. Thirdly, we have found an oligomeric intermediate (probably a heptameric ring-shaped particle) accumulated on the GroEL reassembly pathway. This oligomeric intermediate is unstable and dissociates into monomers when the concentration of essential factors decreases below the minimal one until it interacts with another intermediate to form a stable full-size GroEL particle.

## 2. Results and Discussion

### 2.1. Urea-Induced Unfolding and Refolding of the GroEL Oligomeric Particle

**Figure 1:**
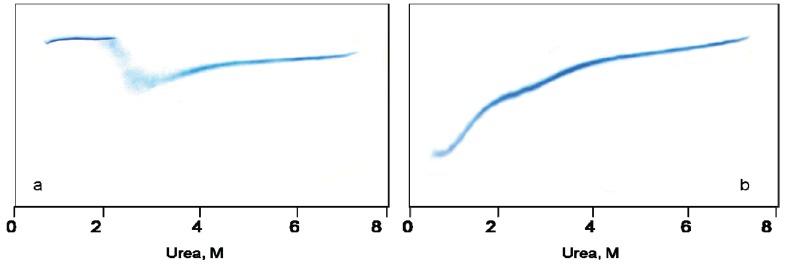
Urea transverse gradient gel-electrophoresis of GroEL. (**a**) Denaturation. Native GroEL (C = 0.5 mg/mL) solution in 20 mM Tris-HCl, pH 7.5 was loaded on the gel; (**b**) Renaturation. Unfolded GroEL (C = 0.5 mg/mL) solution in 20 mM Tris-HCl, pH 7.5, 5 mM β-mercaptoethanol (βME), 5 M urea was applied to the gel.

Figure 1a,b demonstrate urea-induced unfolding of GroEL and its refolding from the urea-unfolded state, as monitored by urea traverse gradient electrophoresis.

**Figure 2:**
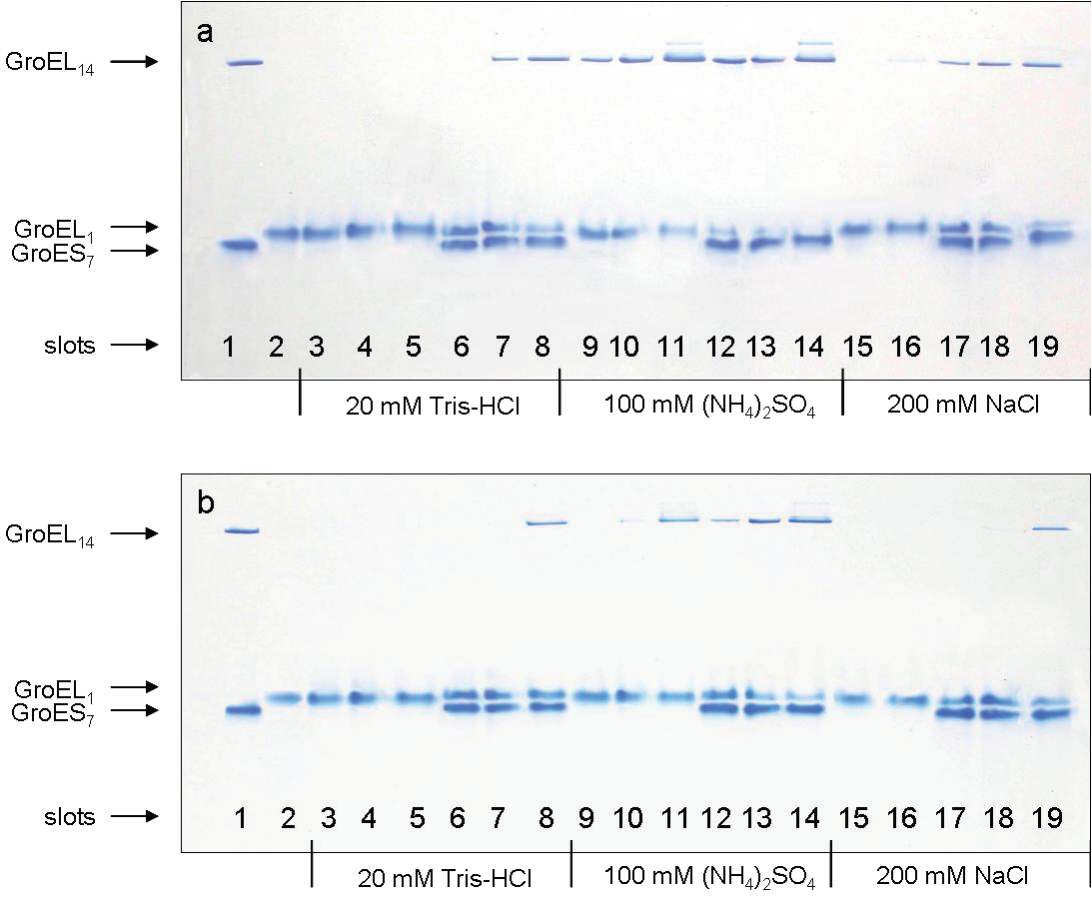
Nondenaturing gel electrophoresis of folded GroEL monomers incubated before applying to the gel during 180 min at 20–22 °C in the presence of Mg-ATP (**a**), Mg-ADP (**b**), and GroES (GroEL_14_:GroES_7_ = 1:2). Slots: 1—the mixture of native GroEL_14_ and GroES_7_; 2—folded monomer GroEL_m_ incubated in buffers both used independently and in the presence of individual ligands; 3—1 mM Mg-nucleotide; 4—10 mM Mg-nucleotide; 5—100 mM Mg-nucleotide; 6, 7 and 8—1 mM Mg-nucleotide, 10 mM Mg-nucleotide and 100 mM Mg-nucleotide in the presence of GroES (all in the buffer 20 mM Tris-HCl, pH 7.5, 5 mM βME, 0.4 M urea); 9, 10 and 11—1 mM, 10 mM and 100 mM Mg-nucleotide; 12, 13 and 14—1 mM, 10 mM and 100 mM Mg-nucleotide in the presence of GroES (all in the buffer 20 mM Tris-HCl, pH 7.5, 5 mM βME, 100 mM (NH_4_)_2_SO_4_, 0.4 M urea); 15 and 16—10 mM and 100 mM Mg-nucleotide; 17, 18 and 19—1 mM, 10 mM and 100 mM Mg-nucleotide in the presence of GroES (all in the buffer 20 mM Tris-HCl, pH 7.5, 5 mM βME, 200 mM NaCl, 0.4 M urea). The concentration of folded monomers in incubation mixtures was 0.4 mg/mL.

**Figure 3:**
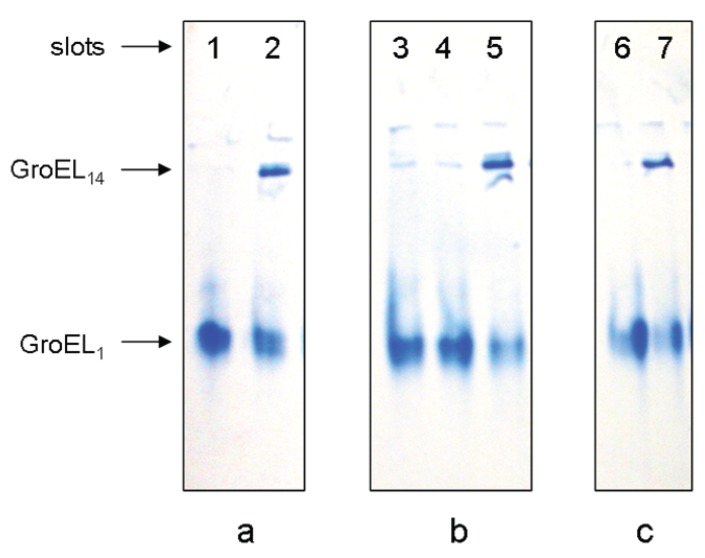
Nondenaturing gel-electrophoresis of folded GroEL monomers incubated before applying to the gel during 180 min at 20–22 °C: (**a**) in the presence of 2 M NaCl or KCl (slot 1), 2 M NaCl or KCl and 10 mM ATP (slot 2); (**b**) in the presence of 20% glycerol (slot 3), 20% glycerol and 10 mM ATP (slot 4), 20% glycerol and 10 mM MgCl_2_ (slot 5); (**c**) in the presence of ammonium sulfate: 100 mM (slot 6) and 1 M (slot 7). The concentration of folded monomers in incubation mixtures was 0.4 mg/mL. Buffer—20 mM Tris-HCl, pH 7.5, 5 mM βME.

**Figure 4:**
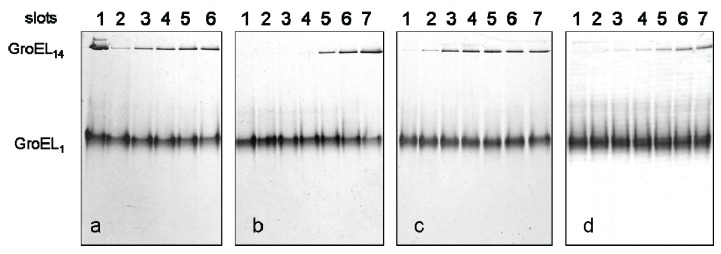
The effect of the factors stimulating GroEL reassembly. Nondenaturing electrophoresis of GroEL monomers after 180 min incubation: (**a**) in 20 mM Tris-HCl, pH 7.5, 5 mM βME, 100 mM (NH_4_)_2_SO_4_, 0.4 M urea, 10 mM ATP and at various concentrations of MgCl_2_ (slots: 2—0.1 mM, 3—0.5 mM, 4—1 mM, 5—5 mM, and 6—10 mM). Slot 1–the mixture of GroEL_14_ and GroEL_1_ in 20 mM Tris-HCl, pH 7.5; (**b**) in 20 mM Tris-HCl, pH 7.5, 5 mM βME, 0.4 M urea, 10 mM MgCl_2_, 10 mM ATP and at various concentrations of (NH_4_)_2_SO_4_ (slots: 1–0 M, 2–1 mM, 3–5 mM, 4–10 mM, 5–50 mM, 6–100 mM, and 7–200 mM); (**c**) in 20 mM Tris-HCl, pH 7.5, 5 mM βME, 0.4 M urea, 100 mM (NH_4_)_2_SO_4_, 10 mM MgCl_2_ and at various concentrations of ATP (slots: 1–0.001 mM, 2–0.01 mM, 3–0.1 mM, 4–0.5 mM, 5–1 mM, 6–5 mM, and 7–10 mM); (**d**) in 20 mM Tris-HCl, pH 7.5, 5 mM βME, 0.4 M urea, 10 mM MgCl_2_ and at various concentrations of glycerol (slots: 1%–14%, 2%–16%, 3%–18%, 4%–20%, 5%–26%, 6%–28%, and 7%–30%). The concentration of folded monomers in incubation mixtures was 0.4 mg/mL.

A change in the protein electrophoretic mobility at a constant charge reflects the change in protein hydrodynamic volume [24,25]. One can see that the change in GroEL hydrodynamic volume upon chaperonin urea-induced unfolding occurs in two distinct stages (Figure 1a). The first one corresponds to a decrease of the protein hydrodynamic volume to a value above 2 M urea that in general reflects disturbance of the GroEL oligomeric structure [20,23,26]. The second one corresponds to an increase of the protein hydrodynamic volume obviously due to further unfolding of the partially unfolded monomers [23,26]. The decrease of the protein hydrodynamic volume during GroEL renaturation from the urea-unfolded state in the absence of protein ligands occurs in two distinct stages (Figure 1b). However, these stages obviously reflect successive refolding of the GroEL subunit domains up to a folded state (~0.5 M urea) possessing a much higher electrophoretic mobility, and hence, a lower hydrodynamic volume than the full-size GroEL particle. Urea-induced unfolding of the folded GroEL monomer (subunit) has the same stages as refolding. These stages manifest themselves through increased hydrodynamic volume (similar to that shown in Figure 1b), as well as through decreased ellipticity at 220 nm, tyrosine fluorescence intensity and anisotropy (not shown). As follows from Figure 1a,b, at 2 M urea (where the GroEL oligomeric structure dissociates) the protein monomers are essentially unfolded, probably due to unfolding of the equatorial domain responsible for intersubunit interactions [26]. The considerable difference in electrophoretic mobility between the full-size GroEL and its folded monomer (Figure 1a,b) allows us to use nondenaturing gel electrophoresis for GroEL reassembly observation. Figure 2 and Figure 3 represent nondenaturing electrophoresis data on incubation of GroEL folded monomers (see Experimental Section) at various combinations of the factors required for their assembly. From these data, we can conclude the following. First, in accordance with literature [16,17,18,19], GroEL reassembly does not occur in the absence of GroEL ligands (Mg^2+^, ADP, ATP, GroES) or in the presence of only one of them at a low or moderate ionic strength (Figure 2, slot 2). Second, specific oligomerization of monomers starts after addition of a certain combination of GroEL ligands dependent on the solvent composition. At a low or moderate ionic strength (~20 mM Tris-HCl or 0.2 M NaCl), even high concentrations of Mg-ATP or Mg-ADP (up to 100 mM) do not stimulate assembly of monomers in the absence of co-chaperonin GroES (Figure 2, slots 3–5, 15 and 16). Ammonium sulfate has a remarkable effect on GroEL reassembly (see also [20,21,22]). In the presence of 0.1 M ammonium sulfate, an appreciable GroEL reassembly is observed in the presence of both Mg-ATP and Mg-ADP (Figure 2, slots 9–11). Besides, Mg-ATP is more efficient than Mg-ADP. Interestingly, addition of a two-molar excess of co-chaperonin GroES (GroEL_14_:GroES_7_ = 1:2) essentially stimulates GroEL oligomerization in all cases. The upper double bands (Figure 2a, lanes 11 and 14) observed at high concentrations of Mg-ATP (100 mM) are probably due to partial association of GroEL tetradecamers (possibly dimerization) in these conditions. Third, the effect of ligands on GroEL reassembly may also be achieved by a certain solvent composition (Figure 3). Mg^2+^ can be replaced by high ionic strength (~2 M NaCl or KCl) of the solvent (Figure 3a). In the presence of 20% glycerol GroEL reassembly occurs in the absence of adenine nucleotides but in the presence of Mg^2+^ only (Figure 3b) (see also [19]). Moreover, in the presence of 1 M ammonium sulfate GroEL reassembly does not require any ligands (Figure 3c) (see also [22]). To learn the concentrations of the ligands (Mg^2+^, ATP) and other factors (ammonium sulfate and glycerol) required for specific GroEL oligomerization, we studied the effect of these factors on GroEL reassembly from monomers in detail. Figure 4 shows the dependence of the GroEL reassembly efficiency on concentrations of various factors. The optimal conditions for GroEL oligomerization have been chosen taking into account the literature reports [16,17,18,19,20,21,22] and the data represented in Figure 2 and Figure 3. These conditions are as follows: 20 mM Tris-HCl, pH 7.5, 100 mM (NH_4_)_2_SO_4_, 10 mM Mg^2+^, 10 mM ATP or 10 mM Mg^2+^ and 20% glycerol. Keeping some oligomerization-responsible factors unchanged and varying others, we found that noticeable oligomerization of GroEL requires the presence of more than 50 mM ammonium sulfate, 0.1 mM ATP, 0.5 mM Mg^2+^ or 10 mM Mg^2+^ and 18% glycerol. Thus, if the GroEL oligomerization reaction starts at the minimal concentrations of the essential factors, it can be stopped by dilution to achieve a concentration of the factors below the minimal value.

### 2.2. GroEL Time-Resolved Reassembly and the Kinetic Intermediate Oligomer

**Figure 5:**
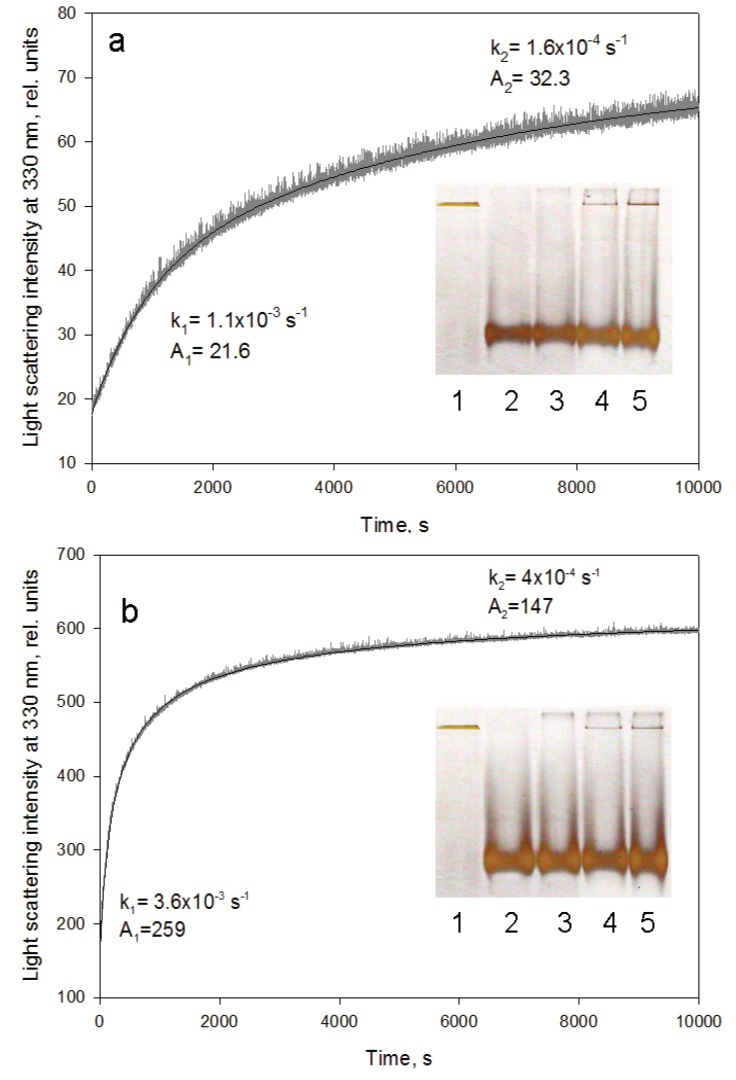
GroEL reassembly kinetics monitored by light scattering at 330 nm. (**a**) in 20 mM Tris-HCl, pH 7.5, 5 mM βME, 10 mM MgCl_2_, 20% glycerol, 0.4 M urea; (**b**) in 20 mM Tris-HCl, pH 7.5, 5 mM βME, 50 mM (NH_4_)_2_SO_4_, 1 mM MgCl_2_, 0.5 mM ATP, 0.4 M urea. Rate constants and amplitudes of the protein oligomerization kinetic phases are shown at corresponding parts of the kinetics. Inserts represent nondenaturing electrophoresis of the products after termination of the oligomerization reaction by 10-fold dilution of the reaction mixture components at various time intervals (slots: 1—full-size GroEL_14_; 2—folded monomer GroEL_1_; 3–500 s; 4–2700 s; 5–5400 s after oligomerization start). The staining of the gels was performed using silver.

**Figure 6:**
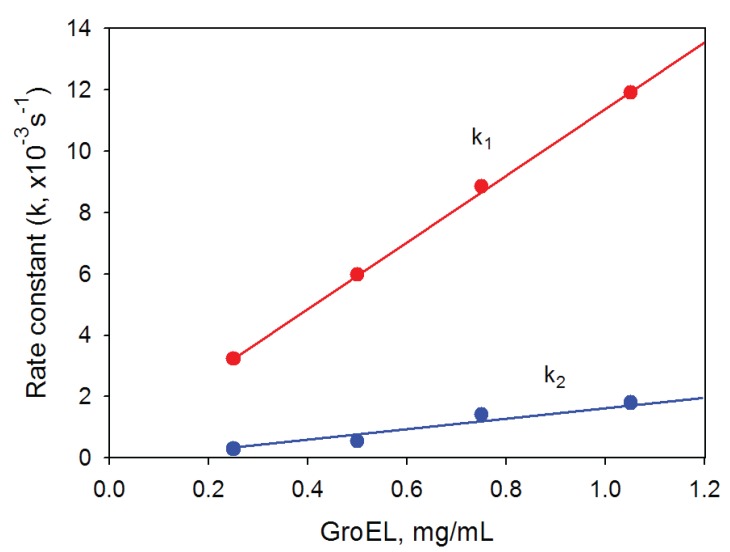
Dependence of the rate constants of GroEL oligomerization kinetic phases on the protein concentration. Buffer: 20 mM Tris-HCl, pH 7.5, 5 mM βME, 100 mM (NH_4_)_2_SO_4_, 0.4 M urea and 10 mM Mg-ATP.

**Figure 7:**
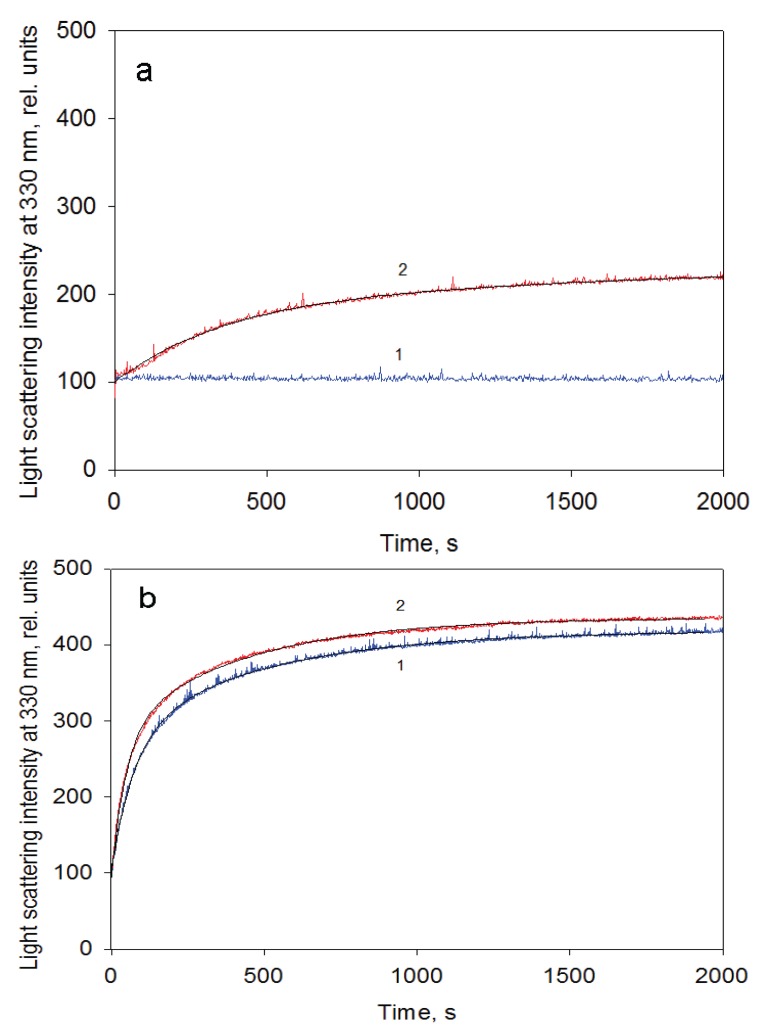
GroEL reassembly kinetics monitored by light scattering at 330 nm in the buffer containing 20 mM Tris-HCl, pH 7.5, 5 mM βME, 100 mM (NH_4_)_2_SO_4_, 0.4 M urea and 10 mM Mg-ATP at a protein concentration of 0.1 mg/mL (**a**) and 1.05 mg/mL (**b**). 1—in the absence and 2—in the presence of GroES (GroEL_14_:GroES_7_ = 1:2).

GroEL oligomerization kinetics can be monitored by increasing light scattering intensity because the large oligomeric particle of GroEL scatters light much stronger than a monomer [17,26]. Figure 5 represents time-resolved oligomerization of GroEL caused by minimal concentrations of the essential factors. As seen, its kinetics appears to be two-exponential and the first phase is an order of magnitude faster than the second (final) one. This suggests accumulation of some intermediate oligomer on the pathway of protein native structure formation. It should be noted that oligomerization kinetics of one-ring structures (for example, mutant GroELSR1 or GroES) is monoexponential (data not shown). Untimely termination of the GroEL oligomerization reaction by dilution of the reaction mixture to essential factor concentrations below their minimal values and an analysis of the reaction products by nondenaturing electrophoresis revealed no intermediate oligomers between the monomers and the full-size GroEL particle (inserts in Figure 5). The upper band at the beginning of the gel (inserts in Figure 5) may correspond to partial nonspecific aggregation of GroEL monomers upon refolding initiation. Equilibrium studies of GroEL unfolding-refolding revealed no noticeable accumulation of intermediate oligomers [16,19,20,22]. However, in the presence of a substrate protein an intermediate GroEL oligomer (probably heptamer) was shown to be stabilized at moderate concentrations of urea [27]. Our data also show that a kinetic intermediate oligomer accumulated on the GroEL reassembly pathway could be a heptamer. Firstly, the linear dependences of the rate constants of GroEL oligomerization kinetic stages on the protein concentration have essentially different slopes (Figure 6). The increase in the rate of the first stage (presumably reflecting assembly of monomers in an intermediate oligomer) is sevenfold that of the last stage (apparently corresponding to formation of the full-size GroEL particle). This result permits suggesting that molar concentrations of the molecules interacting on these kinetic stages are different, and on the first stage, the molar concentration of interacting molecules is essentially larger than that of the last one. Secondly, the rate constant of the first kinetic phase grows when the GroES concentration increases, while the last kinetic phase is much less sensitive to GroES (data not shown). Thirdly, at low GroEL monomer concentrations (less than 0.1 mg/mL) oligomerization occurs only in the presence of GroES, while at large concentrations (~1 mg/mL) the effect of GroES on GroEL oligomerization kinetics is much less pronounced (Figure 7). The data above allow us to suppose that the intermediate oligomer accumulated on the GroEL particle formation pathway is unstable when concentrations of the essential factors are below critical ones, but it stabilizes when it interacts with another one or with GroES to form a full tetradecameric structure. The most preferable candidate on the role of the intermediate oligomer important for GroEL reassembly is a one-ring heptamer capable of binding to GroES and to the substrate protein [28,29,30].

**Figure 8:**
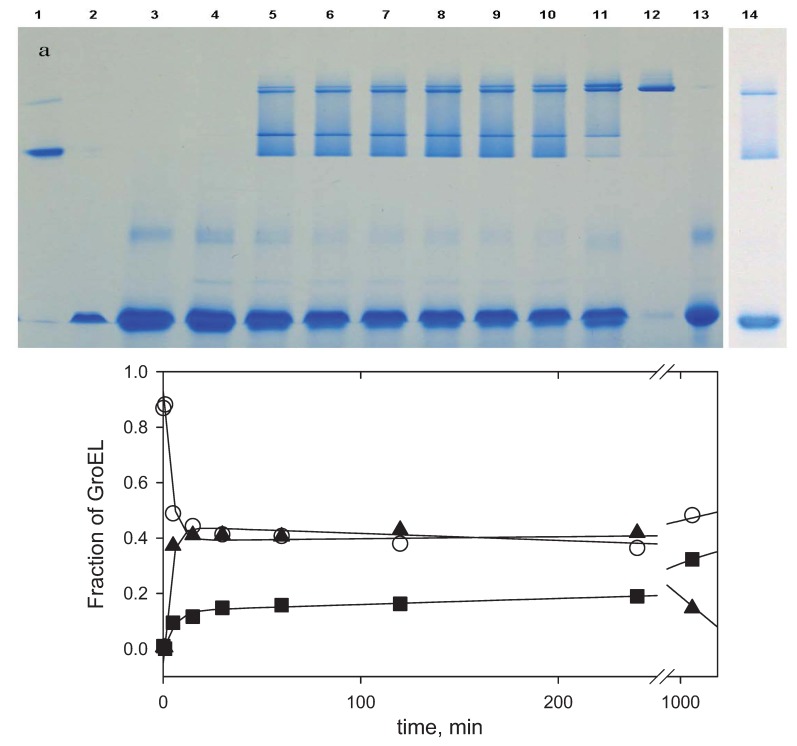
Nondenaturing blue electrophoresis analysis of GroEL oligomerization kinetics: a—the products of GroEL oligomerization kinetics visualized by Coomassie brilliant blue G250 (slots: 1—SR1 one ring mutant of GroEL, 2—GroES heptamer, 3—60 min after GroEL oligomerization reaction was started in the presence of Coomassie brilliant blue G250 dye, 4—1 min, 5—5 min, 6—15 min, 7—30 min, 8—60 min, 9—120 min, 10—240 min and 11—1200 min after the GroEL oligomerization reaction started in the presence of Mg-ATP and two molar excess of GroES, 12—GroEL tetradecamer, 13—GroEL monomer, 14—180 min after the GroEL oligomerization reaction was started in the absence of GroES); b—the time-resolved change of GroEL monomer (○), intermediate oligomer (▲) and full-size GroEL particle (■) during the GroEL oligomerization reaction in the presence of GroES and Mg-ATP. The data were obtained by an analysis of electrophoretic band intensities (Figure 8a) using the computer program TatalLab TL120 1Dv 2009 (Nonlinear Dynamics Ltd., Newcastle upon Tyne, UK). Intensities of the bands corresponding to the intermediate oligomer, the full-size GroEL, and their complexes with GroES were summarized and represented as the intermediate oligomer (▲) and the full-size GroEL particle (■).

**Figure 9:**
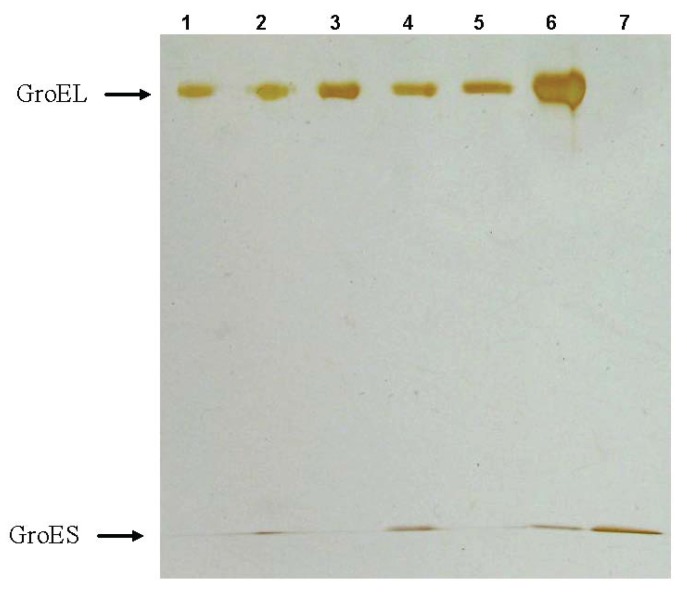
The second-dimension SDS-electrophoresis of the protein bands which are present at nondenaturing blue electrophoresis of GroEL reassembly reaction mixture (see Figure 8). Slots: 1—SR1 mutant of GroEL, 2—complex GroEL_14_ with GroES_7_, 3—GroEL_14_, 4—complex of the intermediate oligomer with GroES_7_, 5—the intermediate oligomer, 6—a GroEL monomer (a few GroES are present because of some difficulty with separation of the corresponding bands– see Figure 8a), 7—the GroES heptamer.

This assumption is confirmed by the analysis of GroEL reassembly kinetics using nondenaturing blue electrophoresis in the presence of Mg-ATP (Figure 8). The blue electrophoresis technique allows us to evaluate the molecular weight of the native protein molecules [31]. Figure 8 represents the blue native electrophoresis analysis of products of the GroEL reassembly reaction in the presence of Mg-ATP and GroES at various time-intervals. From these data one can conclude the following. Firstly, the dye (Coomassie brilliant blue G250) used for the separation and visualization of proteins by blue native electrophoresis inhibits GroEL reassembly when it is added at the reaction start (slot 3). Thus, it is impossible to study GroEL reassembly in the presence of this dye. At the same time, this phenomenon allows preventing protein oligomerization during blue electrophoresis in the presence of Mg-ATP in the gel and electrode buffer. Secondly, with the dye added at various time intervals before GroEL loading on the gel, only a few protein bands were observed. According to the evaluated molecular weight and the corresponding markers, these bands have been attributed (from bottom to top) to GroEL monomer, GroES heptamer (M.W. 67.5 ± 0.3 kDa), GroEL intermediate oligomer (M.W. 412 ± 2 kDa, corresponding to GroEL heptamer), complex of the intermediate oligomer with GroES (M.W. 507 ± 1 kDa), full-size GroEL tetradecamer (M.W. 815 ± 2 kDa), and complex of the full-size GroEL_14_ with GroES_7_ (M.W. 856 ± 2 kDa). The second-dimension SDS-electrophoresis of the corresponding bands (see “Experimental” section) confirms this identification (Figure 9). The time-resolved change of the corresponding conformations during GroEL reassembly in the presence of Mg-ATP and GroES (Figure 8b) shows that there is an appreciable difference in their rates. At the first stage changes of the population of GroEL monomers and the intermediate oligomer were faster than that of full-size GroEL. This is a partial evidence that on the first stage of the GroEL reassembly reaction the intermediate oligomer is formed, while the full-size GroEL particle is formed on the last stage (see Figure 5 and Figure 6).

At least three facts from blue electrophoresis (Figure 8a) are still unclear and may require further study. First, electrophoretic mobility of the GroES heptamer is lower than that of a GroEL monomer in the presence of Mg-ATP, while in the absence of Mg-ATP from the gel and electrode buffer, a GroEL monomer is more slow than GroES (*cf.*Figure 2 and Figure 8a). This may be explained by ATP-to-GroEL monomer binding and acquisition by the latter a more compact conformation [18]; alternatively, the GroES conformation may become less compact in the presence of ATP. Second, the most diffuse bands are between the intermediate oligomer and the full size GroEL tetradecamer. A possible reason for this may be the fast exchange between these GroEL conformations. Third, there is an inexpressive diffuse band between the GroEL monomer and the heptamer (slots 3–10 and 12). Such a band is absent from the electrophoresis run performed without ATP and dye (Figure 2). Probably, this band is a result of stabilization of some intermediate oligomer (likely a trimer) in the presence of ATP and Coomassie dye.

The data obtained can explain the fact that the *GroES* gene precedes the *GroEL* gene within the GroE operon in the *E. coli* genome [7]. Obviously, GroES is necessary for more effective assembly of GroEL, especially at its low concentrations at the initial step of biosynthesis. Moreover, our unpublished kinetic data show that GroES refolding and reassembly occur much faster than those of GroEL and do not require any additional factors.

## 3. Experimental

### 3.1. Solutions

For buffer preparations we used ATP- and ADP-Na-salts, phenylmethylsulfonyl fluoride (PMSF), L-histidine (all “Sigma-Aldrich”, St. Louis, MO, USA); white egg lysozyme, NaCl, KCl, (NH_4_)_2_SO_4_, urea, MgCl_2_, MgCO_3_ (all “Reakhim”, Moscow, Russia); β-mercaptoethanol (βME), Coomassie brilliant blue G250, and Tris (all “Serva”, Heidelberg, Germany). All solutions were prepared using bidistillated water.

### 3.2. Proteins

GroEL and GroES were purified from *Escherichia coli* cells (strain HB101) transformed with multicopy plasmid pGroE4 (GroE operon *E. coli* cloned at the *Eco*R I site of the pACYC 184 vector) according to the published protocols [16,17,18,32]. The cells were harvested by centrifugation and resuspended in 20 mM Tris-HCl (pH 7.5) containing 1 mM EDTA (ethylenediaminetetraacetic acid), 1 mM βME and 0.2 mM PMSF. The cells were disrupted by lysozyme (1 mg per g of cells) and incubated on ice during 10 min. The solution was sonicated five times at 22 kHz during 45 s and the cell debris was removed by centrifugation at 14,000× *g* and 35,000× *g* for 30 min. The supernatant was applied to the DEAE-Toyopearl column (Toyo Soda, Tokyo, Japan) equilibrated with 20 mM Tris-HCl, pH 8.0. The proteins were eluted with linear gradient of NaCl (0 M–0.7 M). GroES was eluted within the first third while GroEL—in the middle of the gradient. Further purification of the proteins was performed using ion-exchange chromatography Mono-Q (Pharmacia, Stockholm, Sweden) and Mono-S (Pharmacia) as well as gel-chromatography on Superose 6 (GroEL) and Superose 12 (GroES). The pET-based construct, including T7SR1, as well as the SR1 purification protocol were kindly given by Dr. Arthur L. Horwich and Dr. Wayne A. Fenton (Yale School of Medicine, New Haven, CT, USA), and are mainly described in [30,33]. The folded monomeric form of GroEL was prepared by size-exclusion chromatography on Superose 6 of the protein incubated in 6 M urea for 40 min [16]. The elution buffer contained 20 mM Tris-HCl, pH 7.5, 100 mM (NH_4_)_2_SO_4_, 5 mM βME, and 10 mM MgCl_2_. The protein purity was controlled by denaturing and nondenaturing electrophoresis as well as by fluorescence and absorption spectra. Refolding and reassembly of GroEL were performed by the dilution of the protein solution in 6 M urea up to 0.4 M urea in the presence of various factors at intensive mixing. In some cases, GroEL refolding was started at 0 °C to diminish the protein nonspecific aggregation with following incubation at 20 °C–22 °C.

### 3.3. Electrophoresis

SDS-PAGE was performed according to the well-known Laemmli method [34]. Urea transverse gradient electrophoresis was performed mainly according to [24,25]. Polyacrylamide gel of the acrylamide concentration gradient from 7% up to 9% contained 380 mM Tris-HCl, pH 8.8, 5% glycerol, 7.5% riboflavin, 0.03% TEMED (tetramethylethylenediamine), 2 mM DTT and the urea gradient from 0 M up to 8 M. Nondenaturing electrophoresis was performed in 9% polyacrylamide gel containing two parts. The separating part contained 9% acrylamide, 380 mM Tris-HCl, pH 8.8, 5% glycerol, 0.1% APS (ammonium persulfate), and 0.05% TEMED. The concentrating part contained 5% acrylamide, 126 mM Tris-HCl, pH 6.8, 0.1% APS, and 0.05% TEMED. The buffer containing 400 mM Tris-HCl, pH 6.8, 50% glycerol and 0.006% bromphenol blue was added in the protein solution before applying to the gel. The electrode buffer contained 1.44% glycerol, 0.3% Tris, pH 8.3–8.5. In special cases, 5 mM ATP and 10 mM MgCl_2_ were added to the gel and electrode buffer.

Blue native electrophoresis allowing evaluation of the native protein molecular weight was performed according to [31] using Coomassie brilliant blue G250 dye. The second-dimension SDS-PAGE was performed after cutting out of the corresponding bands from the blue native electrophoresis and SDS-extraction. In this case, the visualization of the individual polypeptides was executed using silver staining.

### 3.4. Circular Dichroism, Fluorescence and Light Scattering

Far-UV circular dichroism spectra were followed with a Chirascan spectropolarimeter (Applied Photophysics, Leatherhead, UK) at a range of 190–250 nm with a light path length of 0.1 mm. Protein concentration was 0.5 mg/mL. Fluorescence intensity and anisotropy as well as light scattering at 90° were measured with a Cary Eclipse spectrofluorimeter (Varian, Palo Alto, CA, USA) at protein concentrations 0.1–1.0 mg/mL (light scattering) and 0.05 mg/mL (fluorescence). Before the measurements, the protein solution was centrifuged for 10 min at 20,000× *g* and filtered through a 0.22 μm filter (Millipore, Billerica, MA, USA). Kinetic measurements were performed in a standard 1 × 1 × 4 cm quartz cell with magnetic stirrer mixing. The approximation of the kinetic curves was made using the computer program Sigma Plot (Systat Software, Inc., San Jose, CA, USA) and equation f = A_1_exp(k_1_t) + A_2_exp(k_2_t). The protein concentration was determined from the absorption spectra at wavelength of 280 nm using a Cary 100 Bio spectrophotometer (Varian) and extinction coefficients (A^0.1%^_1 cm_): 0.22 for full-size GroEL_14_, 0.19 for the GroEL_1_ folded monomer and 0.14 for GroES [16].

## 4. Conclusions

The data presented here, together with previous information from the scientific literature, allow us to make the following conclusions. First, despite its complex oligomeric and multidomain structure, GroEL is able to adopt the native functionally active conformation from the urea-unfolded state. Second, GroEL urea-induced unfolding starts out from dissociation of its oligomeric structure down to monomers, which in these denaturing conditions are essentially unfolded (Figure 1). Third, GroEL refolding (renaturation) begins with refolding of its subunits up to a conformational state able to oligomerize specifically. For this event, certain conditions are required. We propose that the ability of GroEL subunits to undergo specific oligomerization is based on two major points. On the one hand, it is important to reduce electrostatic repulsion of strong negatively charged subunits (−19 per each, as seen from the amino acid sequence [7]). This results either from Mg^2+^ binding or a high ionic strength (Figure 3). On the other hand, it is necessary to stabilize the structural unit important for intersubunit interactions (our preliminary data suggest that it should be some secondary structure unit). This probably may be achieved through interaction with adenine nucleotides or solvent composition (the presence of ammonium sulfate, glycerol). Fourth, it is necessary to emphasize the key role of co-chaperonin GroES in GroEL reassembly at low chaperonin concentrations (Figure 7) or low and moderate ionic strength (Figure 2). We suppose that this is caused by low stability (low probability of formation) of an intermediate oligomer which is the indispensable condition for assembly of full-size GroEL. Apparently, the GroEL intermediate oligomer becomes stable after its interaction with heptameric GroES (Figure 8 and Figure 9), and therefore, the probability of full-size GroEL formation increases greatly. Thus, GroEL ligands actively participate not only in chaperonin functioning [3,5,9,35] but also in chaperonin folding (see also [36]).
